# Computational Applications: Beauvericin from a Mycotoxin into a Humanized Drug

**DOI:** 10.3390/metabo14040232

**Published:** 2024-04-18

**Authors:** Charbel Al Khoury, Sima Tokajian, Nabil Nemer, Georges Nemer, Kelven Rahy, Sergio Thoumi, Lynn Al Samra, Aia Sinno

**Affiliations:** 1 Department of Natural Sciences, School of Arts and Sciences, Lebanese American University, Beirut Campus, P.O. Box 13-5053, Chouran, Beirut 1102 2801, Lebanon; 2 Department of Natural Sciences, School of Arts and Sciences, Lebanese American University, Byblos Campus, Byblos P.O. Box 36, Lebanon; 3 Department of Agriculture and Food Engineering, Holy Spirit University of Kaslik, Jounieh P.O. Box 446, Lebanon; 4 Division of Genomics and Translational Biomedicine, College of Health and Life Sciences, Hamad Bin Khalifa University, Doha P.O. Box 34110, Qatar; 5 Gilbert and Rose-Marie Chagoury School of Medicine, Lebanese American University, Byblos P.O. Box 36, Lebanon; 6 Department of Computer Science and Mathematics, Lebanese American University, Beirut P.O. Box 13-5053, Lebanon

**Keywords:** mycotoxin, computer-aided drug discovery, natural compounds, beauvericin, repurposing

## Abstract

Drug discovery was initially attributed to coincidence or experimental research. Historically, the traditional approaches were complex, lengthy, and expensive, entailing costly random screening of synthesized compounds or natural products coupled with in vivo validation largely depending on the availability of appropriate animal models. Currently, in silico modeling has become a vital tool for drug discovery and repurposing. Molecular docking and dynamic simulations are being used to find the best match between a ligand and a molecule, an approach that could help predict the biomolecular interactions between the drug and the target host. Beauvericin (BEA) is an emerging mycotoxin produced by the entomopathogenic fungus *Beauveria bassiana*, being originally studied for its potential use as a pesticide. BEA is now considered a molecule of interest for its possible use in diverse biotechnological applications in the pharmaceutical industry and medicine. In this manuscript, we provide an overview of the repurposing of BEA as a potential therapeutic agent for multiple diseases. Furthermore, considerable emphasis is given to the fundamental role of in silico techniques to (i) further investigate the activity spectrum of BEA, a secondary metabolite, and (ii) elucidate its mode of action.

## 1. Beauvericin: A Mycotoxin

Historically, before pure chemicals were synthesized, people used natural compounds for their useful applications in traditional medicine and pest management within a harmonized ecosystem. The secondary metabolites of such natural products are predominantly synthesized by bacteria, fungi, and plants [1]. These molecules possess low molecular weight and exhibit a wide array of chemical structures along with diverse biological activities. Although not required for the development of organisms, they are produced to confer a selective survival advantage [2]. Paradoxically, these compounds (or their derivatives), over the last two decades, have constituted approximately 35% of the FDA-approved drugs being used to control/cure diseases and/or combat microbial infectious agents (bacteria/fungi/viruses) [3]. The assessment of mycotoxins for their wide span of bioactivities has garnered considerable attention [4]. 

**Figure 1:**
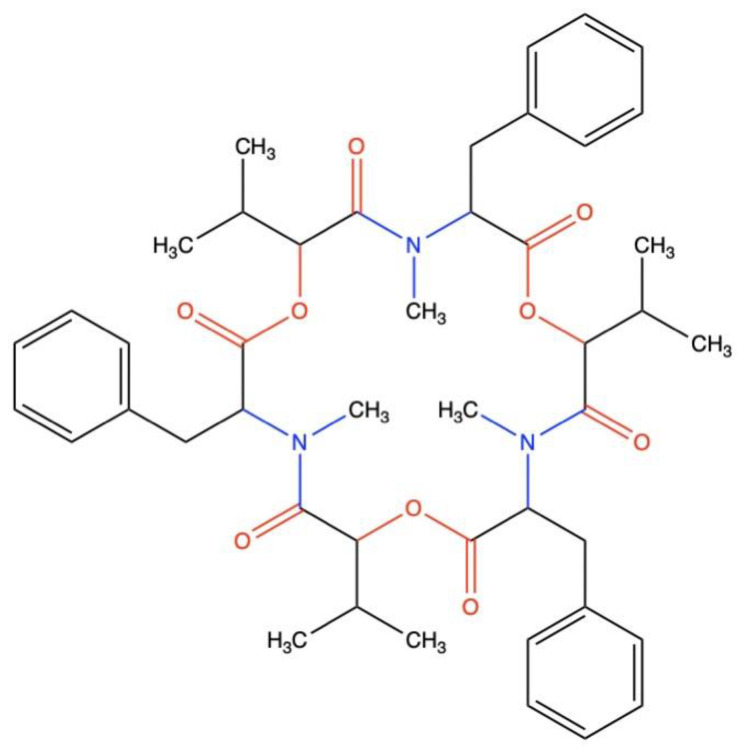
Secondary structure of BEA, a cyclic hexadepsipeptide.

*Beauveria*, a cosmopolitan anamorphic genus of soilborne necrotrophic entomopathogenic fungi, is the most studied biological control agent. Its global distribution in addition to its broad range of target pests have made *Beauveria* the most prominent entomopathogenic fungi [5]. Its ability to cause diseases in insects was initially reported nearly 60 years ago. Since that time, *Beauveria* has been the most studied alternative control agent, and, currently, its conidia form the basis of several commercial insecticides available in the world trade [6]. The first taxonomical characteristic for identifying the *Beauveria* genus was based on the morphology of conidiogenous cells and the conidiogenesis process itself [7]. This traditional approach, however, has always been controversial. In 2011, the first molecular approach entailed a multi-locus phylogenetic approach based on the partial sequences of the protein-coding genes (*RPB1*, *RPB2*, *TEF*), and the B locus nuclear intergenic region (Bloc) [8]. Twelve ex-type species of *Beauveria* were described. Among these, *B. bassiana* was found in different isolates infecting a wide variety of insects and mites including but not limited to Acari, Lepidoptera, Coleoptera, Hymenoptera, Homoptera, Diptera, Hemiptera, and Orthoptera [9,10]. Despite being considered a generalist fungus with no host preference, *B. bassiana* virulence varies considerably among the different strains [11]. Additionally, and in sharp contrast to other entomopathogenic agents, *B. bassiana* can infect its host by ingestion or contact. The most familiar mode of infection is penetration through the host tegument, with infections caused by conidial ingestion and penetration through natural orifices also being reported [12]. The success of establishing a fungal infection depends on the capability of entomopathogenic fungi to overcome the epicuticular fungistatic compounds, cuticle thickness, mineralization, sclerotization, melanization, protease inhibitors, antimicrobial peptides, and cellular/humoral responses [13]. Interestingly, some hypervirulent strains of *B. bassiana* can evade encapsulation and nodule formation by producing secondary metabolites that dampen the host cellular immune response [14]. BEA is the most common secondary metabolite produced by many fungi, including *Beauveria* spp., and is a cyclic hexadepsipeptide molecule belonging to the enniatin antibiotic family [15]. It contains three D-hydroxyisovaleryl and three N-methyl phenylalanyl residues in an alternating sequence (Figure 1). BEA, however, encompasses different biological activities from enniatins produced by *Fusarium* spp., linked mainly to the nature of the N-methylamino acid [16].

## 2. Arthropodpathogenic Activity of Beauvericin

Numerous fungi are known to produce BEA after reaching the hemocoel of their host. Mutant strains of *B. bassiana*, with a knock-out of the *bbBeas* gene responsible for the expression of BEA, exhibit less virulence in their insect hosts [17]. BEA can suppress arthropod cell defenses including those mediated by antimicrobial lipids and phenols, enzyme inhibitors, and proteins [18]. BEA also inhibits the growth of microbial flora in arthropods and other organisms that could compete over limited nutrients [19]. The observations listed above are linked directly to the mechanisms and impact of BEA on the host, which are tightly associated with its molecular characteristics. The first important link is the ionophoric nature of BEA which was first demonstrated in a study back in the 1990s where the authors provided evidence that beauvericin can transport small ions across cytoplasmic membranes [20]. This observation was further supported by showing that BEA could establish a cation-selective channel after incorporating it into mammalian cell membranes [21]. Consequently, the increased intracellular bioavailability of Ca^2+^ decreases the transmembrane potential of the mitochondria, releases cytochrome c, and activates caspase 3, a critical executioner of apoptosis [22]. Thus, BEA can initiate cell death via calcium-dependent pathways [23]. Furthermore, the increase in BEA concentration could breach the nucleus and alter the DNA [24]. BEA can randomly bind to DNA sequences and damaged areas could be fragmented by nucleases. 

Initially, the insecticidal activity of BEA was confirmed against *Aedes aegypti,* a mosquito that can spread dengue fever, and *Calliphora erythrocephala*, flies that are important in forensic entomology [25]. Since then, more details on the insecticidal activity of BEA have become available. BEA can efficiently control the growth of the Colorado potato beetle, *Leptinotarsa decemlineata* [26]. BEA also significantly reduced the fitness of *Schizaphis graminum* by increasing the number of abortive embryos produced by treated females [27], and among 16 fungal metabolites, BEA showed the highest cytotoxicity to the lepidopteran (*Spodoptera frugiperda*) cell line (SF-9) [28]. Moreover, studies on the plant bug *Lygus* spp. provided insight into the potential role that BEA can play against those bugs [29]. The acaricidal activity of BEA has been explored supporting the potential usefulness of BEA as a promising agent for pest management [30]. Under natural conditions, the application of BEA significantly reduced *Tetranychus urticae* populations while showing no phytotoxicity and ecotoxicological risk.

## 3. From Mycotoxin to Humanized Drug

In recent years, BEA has been exploited in the search for new therapeutic options against human pathogens and diseases. Accumulated data from different perspectives further revealed that BEA is being considered as a potential candidate for medicinal research due to its broad range of biological properties. The wide spectrum of BEA biological and biochemical activities was previously studied [15,16,18]. In this review, however, we shed light on studies using computational methods that played a role in rediscovering novel BEA targets. For this, a targeted literature review was conducted in three different databases—ScienceDirect, Web of Science, and PubMed—to detect all potentially eligible peer-reviewed articles using the terms “beauvericin”, “mycotoxin”, “computer-aided drug discovery”, “in silico”, “molecular docking”, and “molecular dynamic simulation”. The relevant literature was critically reviewed and categorized based on the available data.

### 3.1. The Role of BEA in Antimicrobial Resistance

A growing body of literature has revealed that BEA strengthens the efficacy of chemotherapeutics against a wide span of microorganisms. The first line of evidence came from a high-throughput synergy screening that identified BEA as the most potent combination molecule for the treatment of fungal infections [31]. Using a checkboard assay, BEA acted synergistically with ketoconazole, significantly enhancing the therapeutic index against a broad range of fungal pathogens including *Candida albicans*, *C. parapsilosis*, *C. glabrata*, *C. krusei*, *C. tropicalis*, *Aspergillus fumigatus*, *A. niger*, *A. terreus*, *Saccharomyces cerevisiae*, *Cryptococcus neoformans*, and *Fusarium oxysporum.* Given that invasive fungal infections are considered a significant threat to immunosuppressed patients, investigating the efficacy of the synergetic pair against an immunocompromised host was crucial. A combinatorial dosage of BEA (0.5 mg/kg) and ketoconazole (0.5 mg/day) significantly increased the survival of an immunocompromised mouse model infected with *C. parapsilosis*. Moreover, the fixed-dose combination lowered the number of viable fungal cells in several organs, namely, the kidneys, lungs, and brain. The most remarkable finding that emerged from the obtained data was that the observed therapeutic effect was not attained even with a tenfold-higher dose of ketoconazole [31]. Further tests carried out confirmed that BEA renders fungal pathogens more responsive to treatment with fluconazole, another member of the azole antifungal family [32]. Remarkably, this activity is achieved by increasing its accumulation within the cells of *S. cerevisae*, *C. albicans*, *C. neoformans*, and *A. fumigatus* [32]. In parallel, the fungal secondary metabolite was shown to synergize with sub-lethal doses of different pesticides and potentiate their activity against *T. urticae*, the world’s most resistant arthropod [33]. On the other hand, sub-lethal doses of BEA in combination with cyflumetofen, bifenazate, or abamectin significantly suppressed populations of the two-spotted spider mite when compared to single-pesticide treatment. The authors also showed that BEA could not only reverse but also impair the emergence of resistance to xenobiotics in arthropods. Up to this point, the mechanism of action of BEA was still being investigated. Zhang et al. [31] has hinted at the possibility that an efflux pump, in the fungal cell membrane, might be involved. However, our collective understanding of the target protein, interacting residues, and mechanism of binding was limited. It was only after the development and use of recent in silico approaches that the mode of action of BEA was elucidated. 

Multi-drug resistance is generating considerable interest since it is a major treatment impediment [34]. The development of resistance is mainly driven by the misuse and overuse of antimicrobial agents [35]. The emergence and spread of antimicrobial resistance reduce the effectiveness of the available therapeutic arsenal against a wide range of human, animal, and plant pathogens [36]. According to the World Health Organization (WHO) (2020), the development of resistance is threatening global health and development; thus, understanding the underlying resistance mechanisms is important to mitigate the growth and dissemination of resistant populations. In addition to chromosomal mutations, the development of resistance was mainly attributed to the xenobiotic efflux pumps from different families [37]. The most common group of transmembrane pumps is the ATP-binding cassettes (ABC) transporters that are ubiquitously present in prokaryotes, archaea, and eukaryotes [38]. The inward-facing conformation of ABC transporters, formed from two bundles of six transmembrane helices, results in a large internal cavity that binds xenobiotics and chemotherapeutic drugs [39]. The switch from the inward- to the outward-facing conformation will transport the substrate to the extracellular environment [38], reducing, as a result, its bioavailability inside the cell. Even though modern in silico methodologies have become a crucial part of the drug discovery process, showing that BEA can synergistically improve treatment outcomes was a serendipitous finding. In their attempt to counteract the multi-drug resistant phenotype of *C. albicans*, an opportunistic pathogenic yeast, Tong et al. [40] aimed to neutralize the drug efflux pumps *CDR-1* and *CDR-2* that belong to ABC transporters. The swift advancement of genotyping allowed researchers to sequence new proteins in a rapid and significant manner. At a significantly lower pace, scientists are still deciphering and revealing the 3D structure of proteins using X-ray crystallography, solution NMR, electron microscopy, and neutron diffraction. One approach to overcome this would be through computer-aided comparative modeling of proteins. The homology modeling constructs the atomic resolution of a protein of interest by comparing and utilizing the amino acid sequence of its homologue [41]. To generate models of *C. albicans* ABC transporters, Tong et al. [40] used the Alignment Mode algorithm of SWISS-MODEL [42], selecting a known mouse P-glycoprotein crystal structure (PDB: 3G60) as a template. The generated 3D models were utilized for molecular docking, a computer-assisted drug design methodology, to predict the predominant binding mode of ligands [43]. Molecular docking tools explore 3D contact areas between small molecules and macromolecular structures by using a scoring function that ranks molecular binding [44]. AutoDock Vina is one of the most cited tools for predicting protein–ligand interaction [45]. It relies on a Monte-Carlo based iterated search method and multithreading parallelism scheme on multicore machines to improve docking accuracy and speed [45]. Concomitantly, the tool is fast enough to allow virtual screening of publicly available drug libraries enclosing thousands of molecules [45]. Interestingly, among 950 compounds with more than 500 Daltons found in the ZINC15 database, BEA and its analogs had the best affinity to *CDR-1* and *CDR-2* of *C. albicans*. Of note, the secondary metabolite recoded lower binding energy when compared to the co-crystallized inhibitors of the transmembrane proteins QZ59-RRR (cyclic-tris-(R)-valineselenazole) and QZ59SSS (cyclic-tris-(S)-valineselenazole) [40]. The authors here indicated that BEA, and its analogs, can block the substrate-binding cavity of ABC transporters, therefore lowering the translocation of xenobiotics across the membrane. These findings were recently corroborated using the same in silico pipeline for fetching modulators of efflux mediated by ABC transporters [33]. Here, two and four ABC transporters belonging to ABCB and ABCC subfamilies, respectively, were modeled using the mouse P-glycoprotein (PDB: 4M1M) and the bovine multidrug resistance protein 1 (PDB: 6UY0) as templates. The homology models were refined using 3D-refine software by optimizing the hydrogen bonds as well as the atomic-level energy. Molecular docking was performed with a static target crustal structure using MolSoft ICM-pro, the most accurate predictive tool of the binding geometry [46]. The grid was set upon the drug-binding pocket of the ABC transporters where the hydrogen bonding potential, van der Waals potential with carbon-, sulfur-, and hydrogen-like probes, hydrophobic potential, and electrostatic potential were taken into consideration [33]. The internal cavity (~6000 Å^3^) was docked with BEA, cyflumetofen, bifenazate, and abamectin. In addition, the following three drugs having the potential to inhibit ABC transporters were used as positive control: QZ59RRR (PubChem ID: 25195366), QZ59RRR (PubChem ID: 25195367), and verapamil (PubChem ID: 2520). Interestingly, BEA showed lower affinity relative to drugs of interest and the controls when docked against full ABC transporters of *T. urticae*. Accordingly, the authors assumed that BEA might act as a competitive inhibitor of the transmembrane pump and prevent drug transport by inhibiting drug association, increasing its intracellular concentration as a result. Therefore, combinational therapy using BEA and other drugs can be used to overcome multidrug resistance. It is noteworthy that the authors only used molecular docking which in the absence of molecular dynamics simulation and binding free energy will not be enough to determine the stability of the BEA with ABC transporters. Further experimental investigations including long-timescale molecular dynamics are needed to check the binding stability, secondary structure, and conformational changes in the target–drug complexes. A recent study conducted by Al Khoury et al. [47] delved into an investigation aimed at unravelling the mechanisms underlying the action of BEA against efflux pumps in ABC transporters, with a specific focus on ABCG6 within *Leishmania tropica*. The observed upregulation of *ABCG6* upon exposure to miltefosine (ML), a known leishmanicidal, suggests its pivotal role in mediating ML resistance. Consequently, the study conducted detailed molecular investigations into ABCG6 to elucidate the precise interactions between ABC transporter substrates (ML) and inhibitors (BEA), thereby providing invaluable insights into the drug transport process. By employing molecular docking, the study identified specific residues within the transporter, particularly in transmembrane helices 5, 6, 11, and 12, that interact with BEA, contrasting with ML’s interactions primarily with transmembrane helices 6, 10, and 12. These distinct binding patterns shed light on the unique mechanisms through which BEA inhibits efflux and potentially overcomes drug resistance [47]. Furthermore, the investigation unveiled critical conformational changes induced by both ML and BEA binding through comprehensive MD simulations. These simulations not only validate the substantial movements of the apo ABC transporter but also highlight significant conformational alterations triggered by the binding of substrates and inhibitors. The comparison of binding trajectories between ML and BEA underscores BEA’s ability to impede the conformational changes required for efflux initiation by hindering ML binding. Importantly, the study elucidates the underlying factors contributing to BEA’s high affinity for ABC transporters, emphasizing the crucial role of hydrophobic interactions in stabilizing the BEA–transporter complex. Molecular docking analyses further corroborate these findings, suggesting that BEA effectively inhibits ATP hydrolysis and disrupts the conformational cycle necessary for efflux [47]. The comprehensive in silico findings presented offer, for the first time, a deeper understanding of the molecular mechanisms underlying BEA’s action against ABC transporters, shedding light on its potential as a targeted therapeutic agent to combat drug resistance in protozoan pathogens such as *Leishmania tropica*.

### 3.2. The Antiviral Activity of BEA

The first antiviral attributes for BEA were examined on the human immunodeficiency virus type 1 (HIV-1) where the specificity and inhibitory effect of this secondary metabolite was mapped to the integrase mediation of the integration of the virus genome [48]. BEA inhibitory activity was comparable to that of baicalein and robinetin, which were previously designated as being effective antiviral compounds [49]. On top of its anti-inflammatory effects, BEA was also found to significantly inhibit the nuclear translocation of the NF-κB pathway subunits p65 and p50 within the RAW264.7 cells without inducing cell toxicity [50]. The inhibition of the NF-κB pathway, which is involved in the upregulation of genes encoding cytokines such as interleukin (IL)-1 and tumor necrosis factor (TNF)-α, chemokines, and inflammatory mediators such as inducible nitric oxide (NO) synthase (iNOS) (involved in NO production) and cyclooxygenase (involved in prostaglandin E2 secretion) could alleviate the cytokine storm. Furthermore, Severe Acute Respiratory Syndrome Coronavirus-2 (SARS-CoV-2) which caused a rapidly emerging life-threatening disease that was later defined as COVID-19 [51] with variable clinical manifestations from asymptomatic to mild to severe [52]. The patients suffer from influenza-like symptoms, including, but not limited to, fever, cough, sore throat, headache, and olfactory and taste dysfunction [53]. One of the lessons learned from the ongoing SARS-CoV-2 pandemic was the role and importance of computer-aided drug discovery (CADD). In silico techniques have proven indispensable in the SARS-CoV-2 pandemic, from pathogen detection to drug discovery. Whole-genome sequencing of SARS-CoV-2 significantly contributed to characterizing the evolution of the virus in 3D (GenBank: MN908947.3). Given the critical importance of target-based drug discovery, a special emphasis was placed on the 3D structure of the SARS-CoV-2 proteome [54]. Numerous 3D structures of SARS-CoV-2 proteins are publicly available at “https://sars3d.com (accessed on 24 July 2023)” and were considered potential drug targets and candidates for vaccine development [54]. Research groups heavily depend on computer-aided approaches for the discovery of “druggable” proteins which occupy folds that support interactions with a small drug-like molecule. The RNA-dependent RNA polymerase (RdRp), one of the therapeutic targets, plays a vital role in the replication and transcription of SARS-CoV-2 [55]. The enzyme has no homolog in human cells, and so was recognized as an outstanding target for drug development. The inhibitory effects of 99 natural medicinal compounds were computationally investigated against it [56]. To reveal the best binding affinities of the fungal secondary metabolites, AutoDock was used in a combination of blind and targeted molecular docking. A site mapping was first conducted, and the ligands were docked to the whole surface of the protein without considering target pockets. The molecules that attached to the active site with high affinity (>6 kcal/mol), were then selected for further investigations using targeted docking [56]. Among 25 other compounds, BEA met the set criteria and was later docked to the active site of RdRp. BEA established a strong hydrogen bond and hydrophobic interaction with C813 and F812 of the motif E of the palm subdomain (T582 to P620 and T680 to Q815), respectively. The motif contains a higher number of conserved structural elements that are vital for catalytic activity. The palm sub-domain is needed for the recognition of NTPs over deoxy NTPs and catalysis of phosphorylation reaction via metal ion coordination [57]. Other residues involved in the binding with BEA include L758, V587, L602, V588, W598, T586, G597, G596, M601, S592, K593, S814, D865, Y689, and A688. BEA, 18-methoxy cytochalasin J, (22E,24R)- stigmasta-5,7,22- trien-3-β-ol, dankasterone B, and pyrrocidine A showed the lowest binding affinities and were selected for molecular dynamic (MD) studies [56]. MD is a computational technique for investigating the physical movements of molecules over time [58]. The atoms are permitted to “act together” in a time-varying simulation, presenting the future state of the system [59]. MD simulation can elucidate a broad range of biomolecular processes, most importantly, protein–ligand interaction demonstrating the conformation of all the atoms at femtosecond temporal resolution [58]. To understand how BEA was complexed to the RdRp, the MD simulations were performed by GROningen MAchine for Chemical Simulations (GROMACS) [60]. GROMACS is a multipurpose tool to generate MDs by simulating Newtonian equations of motion for systems with macromolecules (protein) and a potential ligand [61]. The cartesian positions of each atom of the protein–ligand complex are stated at each step of the trajectory, generating complex data. Root-mean-square deviation (RMSD), root-mean-square fluctuation (RMSF), radius of gyration (Rg), and hydrogen bonding kinetics are common measures of biomolecules’ spatial variations in an MD simulation. This output is crucial for analyzing protein–ligand interactions and ligand dynamics. The RMSD measures the average distance between the atoms of superimposed proteins and describes the molecule’s overall discrepancy with respect to a reference conformation. Practically, the value is used to analyze the stability of target proteins in combination with the drug. RMSF is an analysis used to measure the rigidity of the polypeptide chain. It calculates the deviations in C-alpha atoms’ coordinates from their average position. The flexibility pattern reflects the location of secondary structure elements in the protein structure. The Rg provides an indication of the mean square distance between the center of gravity and the ends of the protein. The fluctuation in Rg provides a clear indication of the level of compaction (loosening or compression) of the protein. The hydrogen bonding analysis reveals the dynamics of breaking and forming hydrogen bonds between the target protein and the ligand throughout a trajectory. To evaluate the dynamics of the BEA-RdRp complex, a 100 ns MD simulation was generated [56]. The simulation time was sufficient to interpret the protein–ligand interaction as the system reached equilibrium after 10 ns. The RMSD diagram of BEA in complex with RdRp was among the most stable and demonstrated a high degree of complex stability. The BEA-RdRp complex showed elevated fluctuations in amino acids belonging to the N-terminal domain (144–162, 150–158, and 225–237), interface domain (320–326), finger subdomain (494–506), and palm subdomain (564–600). In contrast, reduced fluctuations were notable in amino acids belonging to the finger subdomain (361–390 and 410–432), and the palm subdomain (657–675 and 776–791) [56]. The anchoring of BEA might, as a result, be involved in causing conformational changes in residues that are essential for enzyme activity. In addition, no significant fluctuations in Rg value were detected in the BEA-RdRp complex when compared to the unbound enzyme. Consequently, the 3D conformation of the protein is not compressed nor loosened in the presence of BEA [56]. MD simulations allow admittance to binding free energy changes, leading to the driving force underlying all biological processes. 

The Molecular Mechanics/Poisson Boltzmann Surface Area (MM/PBSA), a compromise between accuracy and speed, is a widely used method for calculating the ligand-binding free energy. This method relies on splitting the molecular mechanic terms and solvation energy to calculate the binding free energy [62]. Fundamentally, this approach is based on calculating the difference between the binding free energy of two solvated molecules in the bound and free states [62]. Among all fungal secondary metabolites, the highest interaction energy was the one with BEA where van der Waals’ forces were the leading contributors. In parallel, hydrogen bonding was considered a minor component of the energy in BEA-RdRp interaction [56]. In a major advance, Al Khoury et al. [63] took a different approach to investigating the anti-viral activity of BEA. Al Khoury and colleagues docked BEA against several promising drug targets of SARS-CoV-2 instead of in-depth screening of the database against a single protein. BEA, surprisingly, showed the ability to interact simultaneously with multiple targets. The emerging idea of “one drug-multiple targets” has the edge over the conventional “one drug-one target” approach. Of particular interest, a higher therapeutic index could result from the cumulative effect of inhibiting multiple proteins and pathways involved in the development of the disease [64]. Given the fact that pharmaceutical agents acting on multiple targets cannot be easily used for in vitro and in vivo studies, computational methods can instead help in identifying the promiscuity of molecules [65]. It is noteworthy that BEA docked with higher affinity to the main protease (3CLpro) and spike glycoprotein (S protein) of the SARS-CoV-2 when compared to its RdRp [63]. The SARS-CoV-2 encoded 3CL-protease is a homodimeric enzyme that processes the cleavage of polyproteins that are translated from the viral RNA [66], including the rod-shaped S proteins that play a fundamental role in viral pathogenesis, evolution, and transmission [67]. From this standpoint, the neutralization of these proteins can impede the viral infection and alleviate the disease symptoms. 

**Figure 2:**
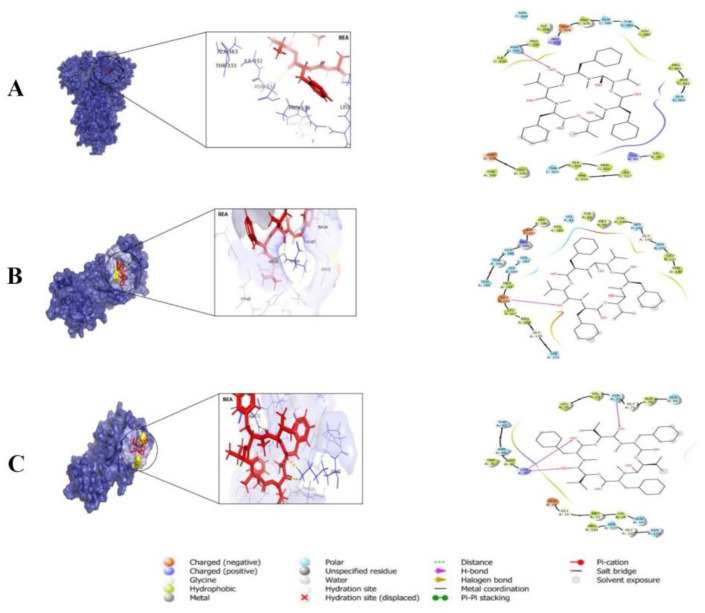
Molecular docking of beauvericin (BEA) with SARS-CoV-2 viral proteins. 3D surface representations of proteins in Blue and BEA is shown in red as well as in 2D diagrams. (**A**) SARS-CoV-2 spike protein. (**B**) Protease: orthosteric site. (**C**) Protease: allosteric pocket.

Additionally, using MolSoft ICM-pro revealed that BEA can strongly bind to H41 (polar interaction) and C145 (hydrophobic interaction), the catalytic dyad residues, along with E166 (H-bond), a vital residue found on the dimerization interface [63]. BEA could also perform allosteric regulation by strongly interacting with an allosteric site, a pocket topographically distinct from the substrate-binding site (Figure 2). The anchoring of BEA to the orthosteric and allosteric sites of 3CL-pro was deemed stable during a 100 ns MD simulation generated by GROMACS [63]. 

Remarkably, molecular docking and MD simulation techniques were integrated to find a new approach for inhibiting 3CL-pro. Further research, however, should be undertaken to screen therapeutic agents against this alternative binding site, away from that of the endogenous agonist. Although allosteric inhibitors have better performance than orthosteric modulators in terms of selectivity and saturability, this approach is still under-investigated. With the S protein, it was speculated that a pocket adjacent to the receptor-binding domain of the spike-ACE2 could be considered an allosteric binding site. However, MD simulations were performed for the BEA–spike complex, and post-MD analysis revealed that the binding of BEA to the pocket did not stir any changes that could render the receptor binding unrecognizable by the substrate [63]. The authors in this study provided clear evidence that molecular docking approaches, although fast, are not able to satisfactorily estimate the predominant binding mode of a ligand with a target protein. Accordingly, additional computationally demanding MD simulations are needed to predict the speed and position of each atom of the considered complex along the sequence of events.

### 3.3. Pharmacokinetics and Drug-Likeness of BEA

A fundamental element moving toward using BEA as a therapeutic agent for humans is to have an insight into its pharmacokinetic properties to elucidate its liberation, absorption, distribution, metabolization, excretion, and toxicity (LADMET) [68]. A high potency does not necessarily imply a good therapeutic index. Moreover, it is essential that high amounts of the molecule reach the target tissue and for a prolonged timespan post-medication [69]. Despite the recently rising interest in determining the pharmacological properties of BEA, the available data regarding the kinetics of the concentration-time profile are limited. Currently, computer-aided prediction of therapeutic LADMET is of particular importance for the development of new drug candidates as it is considered a rapid and accurate process prior to synthesis. SwissADME “www.swissadme.ch (accessed on 13 August 2023), on the other hand, is a freely available tool that compiles robust predictive models for physicochemical properties, pharmacokinetics, drug-likeness, and medicinal chemistry friendliness [70]. BEA was entered in the SMILES list field and submitted to SwissADME calculations. This toolbox facilitates the evaluation of drug-likeness by providing clean molecular and physiochemical characteristics, namely, the molecular weight (MW), number of heavy and aromatic heavy atoms, fraction csp3, number of bonds (rotatable, H-bond acceptor, and H-bond donors), and molar refractivity (Table 1). 

The tool adapts a novel approach based on the summation of tabulated surface contributions of polar fragments instead of the traditional time-consuming calculation of the molecular polar surface area (PSA) that relies on the generation of 3D models and the sum of the surfaces of polar atoms (usually O, N, and attached H), [71]. The in silico predictive software allows for determining lipophilicity during the drug discovery process (Table 2). 

**Figure 3:**
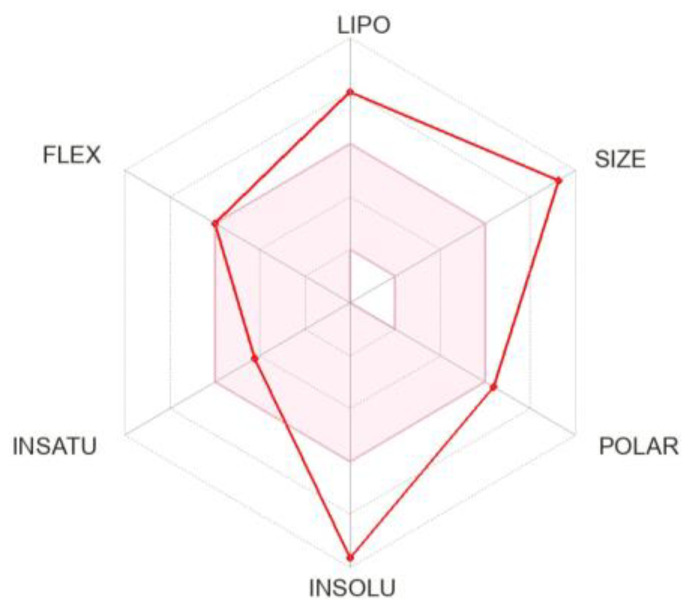
Schematic diagram of Bioavailability Radar for Drug likeness of BEA (lipophilicity: XLOGP3 between −0.7 and +5.0, size: MW between 150 and 500 g/mol, polarity: TPSA between 20 and 130 A2, solubility: log *S* no higher than 6, saturation: fraction of carbons in the sp3 hybridization not less than 0.25, and flexibility: no more than 9 rotatable bonds).

The affinity of a therapeutic agent for a lipid environment is pivotal to the LADMET properties of the drug and a significant contributor to its potency and selectivity. SwissADME gathers five freely available models to evaluate this physicochemical parameter including XLOGP3 [72], WLOGP [73], MLOGP [74,75], SILICOS-IT (http://silicos-it.com, accessed on 24 April 2022), and finally iLOGP [76]. Moreover, the tool provides the consensus log *P*_o/w_ (5.21) considering the arithmetic mean of the values predicted by the five models (Table 2; Figure 3) which shows the high lipophilicity of BEA. In silico prediction of the latter characteristic ties well with previous clinical studies wherein BEA was demonstrated to bioaccumulate in fat-rich tissues [77,78]. These results were further supported by Rodríguez-Carrasco et al. [79] who emphasized the pharmacological profile of BEA and its distribution in serum, urine, muscle, colon, fat, brain, kidney, and liver extracts of mice. The lipophilic nature of BEA is mainly due to the apolar side chains directing out from the outer surface of the molecule [80,81].

Histochemical studies have revealed that BEA can be detected in all biological samples except in urine. It can be thus conceivably hypothesized that this secondary metabolite can diffuse across all biological membranes. Taken together, a high pharmacological activity of BEA might be assumed. Solubility—the dissolution ability of a xenobiotic, the solute, in another substance, the solvent—is another vital characteristic that must be tackled in the pursuit of new potential medicines. SwissADME relied on three different approaches to predict the water solubility of BEA (Table 3). 

**Figure 4:**
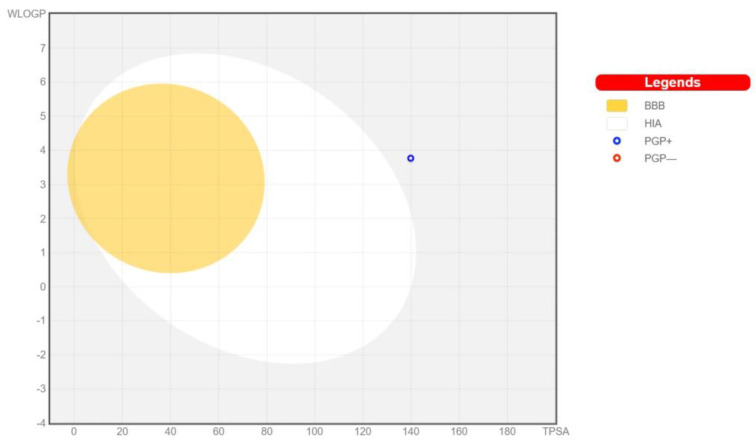
Schematic representation of perceptive evaluation of passive gastrointestinal absorption and Bbain penetration with BEA in the WLOGP-versus-TPSA using BOILED-Egg. The white region is for high probability of passive absorption by the gastrointestinal tract, and the yellow region (yolk) is for high probability of brain penetration. Yolk and white areas are not mutually exclusive. In addition, the points are colored in blue if predicted as actively effluxed by P-gp (PGP+) and in red if predicted as non-substrate of P-gp (PGP−).

The first one is a topological approach considered as an implementation of ESOL model (Solubility class: Log S Scale: Insoluble < −10 poorly < −6 moderately < −4 soluble < −2 very < 0 < highly) [82]. The second predictor is a topological method [83] (Solubility class: Log S Scale: Insoluble < −10 poorly < −6 moderately < −4 soluble < −2 very < 0 < highly). These two approaches avoid the melting point parameter and therefore, differ from the general solubility equation [84]. In addition, they exhibit a significant linear correlation between predicted and experimental scores (R^2^ = 0.69 and 0.81, respectively) [70]. The third one is a fragmental method developed by SILICOS-IT (Solubility class: Log S Scale: Insoluble < −10 poorly < −6 moderately < −4 soluble < −2 very < 0 < highly) “http://silicos-it.com (accessed on 24 June 2023). The linear correlation coefficient of this approach corrected by molecular weight is R^2^ = 0.75. The predicted decimal logarithm of the molar solubility in water were −9.64 (poorly soluble), −11.23 (insoluble), and −10.22 (insoluble) for Log *S* (ESOL), Log *S* (Ali), and Log *S* (SILICOS-IT), respectively (Table 3; Figure 2). Once again, this computational technique was suitable for the prediction of another important physicochemical descriptor. The low water solubility of BEA was first reported by Hamill et al. [85]. This correlates satisfactorily with the observations reported later by Thakur et al. [78] and further supports the idea of the low dissolution of BEA in water. Moreover, this low aqueous solubility was attributed to the lack of chargeable groups [86]. Being at the center of the current drug discovery paradigm, computer modeling is widely used to elucidate the structure–property relationship as well as the pharmacokinetics of the molecule of interest. This computational pharmacological approach is now allowing scientists and the pharmaceutical industry to discover shortcuts and simulate virtually “what the biosystem does to the xenobiotic” [87]. The Brain or IntestinaL EstimateD permeation predictive model (BOILED-Egg) is a rapid graphical method to forecast human gastrointestinal (GI) absorption and blood–brain barrier (BBB) permeation. The evaluation plot involves the yolk and the white, the physicochemical spaces for high probable BBB permeation and GI absorption, respectively [70]. The oral route is, by far, the most common, cost-effective, and easiest route for drug administration. Therefore, assessing the drug GI absorption kinetics is of paramount importance in drug discovery. A major drawback in enteral medication is the necessity of higher doses when compared to other routes because of the first-pass metabolism exhibited by the liver before reaching the systemic circulation. According to the white of the BOILED-Egg, BEA has a low extent of absorption by the GI tract (Figure 4). 

This substantiates previous in vivo findings wherein a low plasma concentration of BEA was detected in pig blood after administration of an intra-gastric bolus [88]. Similarly, the intestinal bioavailability of BEA was measured in Caco-2 cells, a gastrointestinal barrier absorption in vitro model [89]. BEA bioavailability varied between 50.1% and 54.3%, with relatively low values when compared to other structurally related cyclic hexadepsipeptides [90]. As expected, the hydrophilic milieu of the GI tract impedes the absorption of lipophilic xenobiotics such as BEA. Most likely, the molecule will be directly absorbed via the hepatic portal vein and/or the lymphatic system after oral administration. The epithelial-like tight junctions within the brain capillary endothelium are known to inhibit the influx of most pharmaceuticals. Serving as the “strenuous janitor” of our central nervous system, this blood–brain barrier (BBB) is considered a major drawback in brain-targeting drugs. The in silico tool showed that BEA is not brain-penetrant (outside the yolk) and is subject to active efflux (blue dot) (Figure 3). The in silico prediction is not in line with previous experimental results. In fact, a study conducted by Taevernier et al. [91] investigated the BBB transport kinetics of BEA using an in vivo mice model. Due to its lipophilicity, the author showed that BEA can cross the BBB as it exerts a high influx rate to the brain. Equally critical is the assessment of the pharmacodynamics of the drug with cytochrome P450 (CYP). It was previously demonstrated that five chief isoforms of this enzyme (CYP1A2, CYP2C19, CYP2C9, CYP2D6, CYP3A4) can reduce the therapeutic index of 90% of drugs through metabolic transformation [92]. The CYP family is known as a major cause of unexpected drug–drug interaction due to shared metabolic pathways [93]. To determine which CYP isoform is affected, SwissADME endorses a support vector machine algorithm (SVM) for the dataset of known CYP inhibitors/non-inhibitors [70], and BEA was shown to be a non-inhibitor of CYP important isoenzymes (Table 4). 

Contrary to the in silico predictions, using rat and human liver microsomes, Mei et al. [94] revealed that BEA is a potent inhibitor of the CYP isoenzymes (Table 4). The external barriers, including the skin, are our first line of defense which makes it mechanically difficult for pathogens to enter the body’s tissues. Nevertheless, skin permeability for some molecules rendered the skin barrier an interesting target in therapeutic drug delivery. This computational approach also proposes one linear model for skin permeation [95]; the more negative the log Kp (with Kp in cm/s), the less skin-permeant the molecule. The in silico approach predicted a low skin permeability of BEA (log Kp = −5.10 cm/s), whereas an in vivo dermal kinetic study demonstrated that BEA, with high lipophilic characteristics, could penetrate through the skin [96]. Furthermore, the detected BEA concentration in the epidermis was 21 to 46 times higher than that observed in the dermis. Taevernier et al. [96] noticed that the outermost layer of dead cells called stratum corneum acts as a “partial barrier” to many potentially harmful chemicals, including the cyclic hexadepsipeptides. In addition, the transdermal penetration was only 13 to 20 times higher in the case of skin from which the stratum corneum was removed by tape stripping [96]. Given this transdermal behavior, it can be suggested that dermatological formulations of BEA are more attractive than oral administration, especially in the treatment of cutaneous diseases. For that, the development of topical medications based on similar hexadepsipeptides to treat dermatological diseases such as psoriasis, eczema, and skin cancer is currently being exploited [97]. 

The outcome of the in silico drug-likeness analysis can be governed by five rules. First, Lipinski’s rule of five includes H-bond donors > 5; H-bond acceptors > 10; molecular weight > 500 g/mol; and LogP (lipophilicity) > 5) [98]. Second, Ghose’s rule includes 160 < molecular weight (g/mol) < 480; −0.4 < LogP < 5.4; 20 < atom count < 70; 40 < molar refractivity < 130; and polar surface area (Å^2^) < 140, [99]. Third, Veber’s rule includes rotatable bonds ≤ 10; and topological polar surface area (Å^2^) ≤ 140 [100]. Fourth, Egan’s rule includes LogP ≤ 5.88; and the total polar surface area (Å^2^) ≤ 131 [101]. Fifth, Muegge’s rule includes 200 ≤ molecular weight (g/mol) ≤ 600; −2 ≤ LogP ≤ 5; total polar surface area (Å^2^) ≤ 150; number of rings ≤ 7; number of carbons > 4; number of heteroatoms > 1; number of rotatable bonds ≤ 15; H-bond acceptors ≤ 10; and H-bond donors ≤ 5 [102] (Table 5). 

These rules imply that small molecules that fulfill the criteria, within a particular range, are “drug-like” and possess good absorption and permeation. It is noteworthy that BEA fails to comply with Lipinski’s, Ghose’s, and Muegge’s rules (Table 5). The criterion that was not brought to completion is the molecular weight of BEA (783.95 g/mol) and, therefore, lower chances of bioavailability could be anticipated after oral administration. It is important to highlight the fact that many currently available drugs or drug candidates are administered parenterally [103]. Notably, an increasing number of therapeutics that violate the rule of five are currently in clinical trials. Moreover, a decent number of “beyond the rule of five” molecules are now FDA-approved and being prescribed by physicians [103,104]. 

## 4. Conclusions

In this review, we summarized how in silico strategies guided the drug repositioning of BEA, a secondary metabolite, to a molecule that inhibits multidrug efflux by neutralizing the activity of ABC transporters. This is considered as a powerful approach to abrogate ABC transporter-mediated drug resistance and enhance the efficacy of existing drugs. In addition, we provided an overview of the utility of computational tools to speed up the complex drug discovery process and to better prepare and respond to future pandemics. The utilization of molecular docking and MD simulations underscored BEA as a potential therapeutic against SARS-CoV-2. Computational analyses reveal BEA’s strong affinity for key viral proteins, including the main protease and spike glycoprotein. These findings suggest BEA’s potential to hinder viral infection and alleviate COVID-19 symptoms, highlighting the importance of computational methods in identifying promising drug candidates.

Ultimately, computer-aided drug design possesses the capability to predict the physicochemical properties and structural features of the molecule of interest. Consequently, it plays a pivotal role in optimizing drug design and filtering through the use of exclusionary criteria to sift through drug candidates, especially those with undesirable pharmacokinetic properties. 

Here, we demonstrated how computational techniques were crucial in understanding the LADMET profile of BEA as well as elucidating BEA’s drug-likeness, solubility, permeability, and adherence to pharmacokinetic rules, facilitating its potential as a therapeutic agent. All things considered, using computational methods has become an invaluable tool and should be applied at various stages of drug discovery to expedite, filter, and streamline the drug discovery process.

In conclusion, while in silico methodologies represent invaluable assets in modern drug discovery endeavors, their utility is complemented by a recognition of their inherent limitations. One such limitation lies in the simplification of biological systems inherent in computational models, which may not faithfully capture the complexities of real-world interactions. Assumptions underlying these methods, particularly in docking simulations, can potentially oversimplify molecular interactions, thereby compromising the accuracy of predictions. Moreover, the reliance on existing biological knowledge introduces a degree of bias and may hinder the discovery of novel drug–target interactions. The indispensable need for experimental validation underscores the necessity for bridging computational predictions with empirical data to ensure the robustness and reliability of in silico findings. Furthermore, the computational resources and time required for certain simulations can pose practical constraints, limiting accessibility to researchers with restricted computational infrastructure. Additionally, the dependence on scoring functions in molecular docking simulations introduces uncertainties regarding the accurate prediction of binding affinities and the discrimination between active and inactive compounds. Moreover, the scope of applicability of in silico methods may be constrained by the complexity of certain biological systems or the nature of specific drug–target interactions. For instance, challenges arise when dealing with highly flexible ligands or multi-protein systems where traditional computational approaches may struggle to provide accurate predictions. For instance, density gradient-based approaches offer novel avenues for studying drug–protein interactions, presenting innovative solutions to address challenges encountered with in silico methods. These techniques leverage the principles of density gradient centrifugation to separate and characterize drug–protein complexes based on their buoyant densities, providing insights into their composition, stability, and dynamics.

Despite these challenges, it is imperative to acknowledge that in silico methods remain indispensable tools in drug discovery, offering unparalleled capabilities in screening vast chemical libraries, predicting molecular interactions, and optimizing lead compounds. When used judiciously and in conjunction with experimental validation, these computational techniques contribute significantly to accelerating the drug discovery process, reducing costs, and minimizing experimental efforts. Consequently, the integration of computational and experimental approaches represents a synergistic paradigm that maximizes the efficiency and success rate of drug discovery campaigns, ultimately facilitating the development of novel therapeutic agents to address unmet medical needs.

## Figures and Tables

**Table 1:** Computed physicochemical properties of BEA.

Physicochemical Properties	
Formula	C_45_H_57_N_3_O_9_
Molecular weight	783.95 g/mol
Num. heavy atoms	57
Num. atom. heavy atoms	18
Fraction Csp3	0.47
Num. rotatable bonds	9
Num. H-bond acceptors	9
Num. H-bond donors	0
Molar Refractivity	228.14
TPSA	139.83 Å^2^

**Table 2:** Computed lipophilicity of BEALipophilicity.

Lipophilicity	
Log Po/w (iLOGP)	5.29
Log Po/w (XLOGP3)	8.42
Log Po/w (WLOGP)	3.77
Log Po/w (MLOGP)	3.14
Log Po/w (SILICOS-IT)	5.43
Consensus Log Po/w	5.21

**Table 3:** Computed water solubility characteristics of BEAWater solubility.

Water Solubility	
Log S (ESOL)	−9.64
Solubility	1.78 × 10^−07^ mg/mL; 2.27 × 10^−10^ mol/L
Class	Poorly soluble
Log S (Ali)	−11.23
Solubility	4.67 × 10^−09^ mg/mL; 5.96 × 10^−12^ mol/L
Class	Insoluble
Log S (SILICOS-IT)	−10.22
Solubility	4.68 × 10^−08^ mg/mL; 5.97 × 10^−11^ mol/L
Class	Insoluble

**Table 4:** Computed pharmacokinetics parameters of BEA.

Pharmacokinetics	
GI absorption	Low
BBB-permeant	No
P-gp substrate	Yes
CYP1A2 inhibitor	No
CYP2C19 inhibitor	No
CYP2C9 inhibitor	No
CYP2D6 inhibitor	No
CYP3A4 inhibitor	No
Log Kp (skin permeation)	−5.10 cm/s

**Table 5:** Computed druglikeness rule and bioavailability of BEA.

Druglikeness	
Lipinski	No; 2 violations: MW > 500, NorO > 10
Ghose	No; 3 violations: MW > 480, MR > 130, #atoms > 70
Veber	Yes
Egan	No; 1 violation: TPSA > 131.6
Muegge	No; 2 violations: MW > 600, XLOGP3 > 5
Bioavailability Score	0.17

## Data Availability

No new data were created or analyzed in this study. Data sharing is not applicable to this article.
